# Construction and evaluation of a nomogram for predicting survival in patients with lung cancer

**DOI:** 10.18632/aging.203974

**Published:** 2022-03-23

**Authors:** Jin Ouyang, Zhijian Hu, Jianlin Tong, Yong Yang, Juan Wang, Xi Chen, Ting Luo, Shiqun Yu, Xin Wang, Shaoxin Huang

**Affiliations:** 1 Laboratory of Precision Preventive Medicine, Medical School, Jiujiang University, Jiujiang, Jiangxi 332000, PR China; 2 Jiangxi Provincial Key Laboratory of Preventive Medicine, Nanchang University, Nanchang 330006, PR China; 3 SpecAlly Life Technology Co. Ltd., Wuhan, Hubei 430075, PR China; 4 Laboratory Department, Jiujiang University Clinical Medical College, Jiujiang University Hospital, Jiujiang, Jiangxi 332000, PR China; 5 School of Public Health, Qingdao University, Qingdao 266100, PR China

**Keywords:** lung cancer, prognosis, risk score, nomogram, gene expression omnibus

## Abstract

Background: Lung cancer is a heterogeneous disease with a severe disease burden. Because the prognosis of patients with lung cancer varies, it is critical to identify effective biomarkers for prognosis prediction.

Methods: A total of 2325 lung cancer patients were integrated into four independent sets (training set, validation set I, II and III) after removing batch effects in our study. We applied the microarray data algorithm to screen the differentially expressed genes in the training set. The most robust markers for prognosis were identified using the LASSO-Cox regression model, which was then used to create a Cox model and nomogram.

Results: Through LASSO and multivariate Cox regression analysis, eight genes were identified as prognosis-associated hub genes, followed by the creation of prognosis-associated risk scores (PRS). The results of the Kaplan-Meier analysis in the three validation sets demonstrate the good predictive performance of PRS, with hazard ratios of 2.38 (95% confidence interval (CI), 1.61–3.53) in the validation set I, 1.35 (95% CI, 1.06–1.71) in the validation set II, and 2.71 (95% CI, 1.77–4.18) in the validation set III. Additionally, the PRS demonstrated superior survival prediction in subgroups by age, gender, p-stage, and histologic type (*p* < 0.0001). The complex model integrating PRS and clinical risk factors also have a good predictive performance for 3-year overall survival.

Conclusions: In this study, we developed a PRS signature to help predict the survival of lung cancer. By combining it with clinical risk factors, a nomogram was established to quantify the individual risk assessments.

## INTRODUCTION

Lung cancer remains a highly lethal disease, with a 5-year survival rate of only 19% [1, 2]. Despite progress in treatment strategies, due to late diagnosis, the high mortality rate of lung cancer patients did not drop sharply [2]. Therapy of non-small cell lung cancer (NSCLC) patients has evolved over the past few years with the incorporation of targeted therapy and immune therapy. These changes have increased the importance of prognostic and predictive biomarkers [3]. However, various disease outcomes have been identified in patients with similar clinical and pathological features, suggesting that the current clinical prognostic factors may be insufficient to consistently predict individual clinical outcomes [4].

With the development of high-throughput technology, RNA-sequencing (RNA-seq) has been broadly used to identify more novel biomarkers in lung cancer research [5]. Talip Zengin et al. used the TCGA database to identify 12 risk genetic features to predict prognosis in patients with lung adenocarcinoma (LUAD), with the AUC values of 0.479 at 1 year, 0.571 at 2 years, 0.622 at 5 years, and 0.676 at 10 years [6]. Shicheng Li et al. identified eight candidate genes related to survival in LUAD. Zuo, S et al. identified the six-gene signature with AUC values of more than 0.650 for 1, 2, 3, 4, and 5-year overall survival (OS) in LUAD [5, 6]. However, the suggested signatures lack consistency among studies and provide limited prognostic information, partially due to the limited sample size and technical factors [7, 8]. To date, all studies that have been executed in an attempt to find prognostic biomarkers for clinical use have failed to achieve higher sensitivity and specificity or are not easily to be validated in external cohorts with relatively small numbers [9–12].

In this study, an eight-gene prognostic signature was identified by evaluating the prognostic value of the related genes to formulate a prognosis-related risk score (PRS). Moreover, we incorporated genes signature and clinical parameters to establish a novel promising prognostic nomogram model with more accurate predictive ability than clinical risk factors for lung cancer patients. Our work may provide a reference for clinicians to formulate more rational treatment strategies, analyze the pathways and possible mechanisms that may affect the prognosis-related lung cancer, and evaluate the differentiation, calibration and clinical value of the model.

## MATERIALS AND METHODS

### Dataset preparation and samples information collection

**Figure 1:**
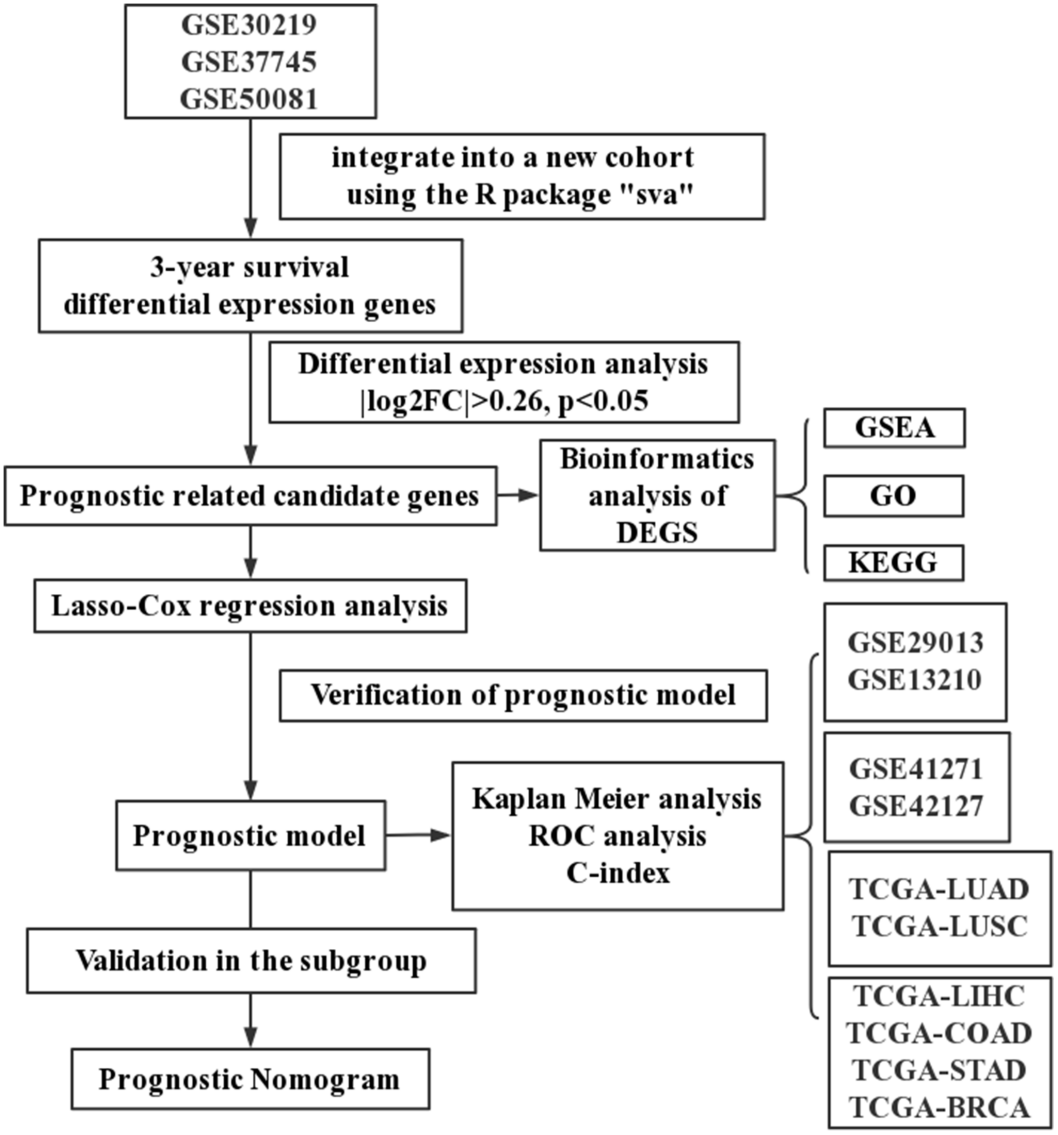
A schematic flowchart for analyzing prognosis-related risk score in lung cancer.

As shown in Figure 1, four sets of subjects were enrolled for preliminary and further verification of screened prognostic biomarkers. In this study, a total of 2325 lung cancer patients who had clinical and follow-up annotations were included in a training set and three validation sets. Of these, 651 patients in the training set came from GSE30219, GSE37745 and GSE50081 (Affymetrix HG-U133 Plus 2.0 Array). These microarray datasets were downloaded from the gene expression omnibus (GEO) database (http://www.ncbi.nlm.nih.gov/geo/) and normalized using a robust multichip average (RMA) algorithm. After batch effects were removed using the combined association test (COMBAT) empirical Bayes method in the surrogate variable analysis (SVA) package, these datasets containing 651 qualified lung cancer patients that were further integrated into a new cohort as the training set. Moreover, similar datasets were processed on the same platform using the identical normalization method and log2 transformation. Validation set I contained a total of 259 lung cancer patients from GSE29013 and GSE31210, and validation set II included 441 lung cancer patients from GSE41271 and GSE42127. The fragments per kilobase per million (FPKM)-normalized RNA-seq data of 494 LUAD and 480 lung squamous cell carcinoma (LUSC) patients retrieved from The Cancer Genome Atlas (TCGA) were integrated as validation set III. We excluded patients with an overall survival (OS) of less than 30 days or with a vague or absent vital status.

In addition, we also established the specificity validation sets of four other cancers, including liver hepatocellular carcinoma (TCGA-LIHC), colon adenocarcinoma (TCGA-COAD), stomach adenocarcinoma (TCGA-STAD), and breast invasive carcinoma (TCGA-BRCA). Supplementary Table 1 shows the dataset information within each cohort.

### Identification of survival-related gene and functional enrichment analysis

The average OS period of the patients in the training set was approximately 3 years, which is a critical point in time. DEGs were identified between alive and deceased subjects within 3 years using the linear models for microarray (LIMMA) package, with difference multiples >1.2 and *p-*value < 0.05 and were selected for further analysis. Furthermore, we performed Gene Ontology (GO) and Kyoto Encyclopedia of Genes and Genomes (KEGG) analysis using the R package clusterpro filer at the level of *p*-value < 0.05 and false discovery rate <0.05. Additionally, the gene set enrichment analysis (GSEA) algorithm in R package gene set variation analysis (GSVA) was used to evaluate the biomarker performance in the training sets retrieved from the Molecular Signature Database (MSigDB) [13].

### Candidate selection and signature establishment

The least absolute shrinkage selection operator (LASSO) algorithm was used to identify the 306 DEG candidate genes with the best survival prediction features in the training sets. Subsequently, we performed multivariate Cox regression analysis based on the results of LASSO analysis. Cox proportional risk regression models were used to assess the importance of each candidate for OS. PRS were calculated as follows: PRS = exp_gene1_ × β_gene1_ + exp_gene2_ × β_gene2_ + ⋯ + exp_geneN_ × β_geneN_ by weighting normalized gene expression values according to their Cox coefficients.

Study subjects in each dataset were divided into high- and low-risk groups according to the cut-off points of median risk scores. Kaplan-Meier (K-M) survival curves and time-dependent receiver operating characteristic (survival-ROC) were conducted to evaluate the prognostic value of the risk score model. The higher the calculated C-index, the more precise the prediction.

### Validation of the prognostic signature

In the training set, a stratification analysis was performed to determine whether the prognostic signature could accurately predict patient survival in different clinical factor subgroups. The model's performance was further evaluated using the independent validation set. In addition, the specificity of the model was tested in four other vital cancers. Gene expression data from different sets were adjusted individually by subtracting the median expression value after log2 transformation.

The PRS was combined with clinically informative variables to create a multivariate cox regression model (complex model) and a nomogram to visualize the predicted outcome for each patient. Additionally, the Hosmer-Lemeshow test was used to validate calibration curves that were established to improve the accuracy of nomogram prediction [14]. To accomplish this, we calculated the total score derived from the established nomogram for each patient in the validation set and generated a calibration curve with Cox regression [15].

### Statistical analysis

IBM SPSS Statistics 26 (IBM Corp., Armonk, NY, USA), GraphPad Prism 7.0 (GraphPad Software Inc, San Diego, CA, USA), the EmpowerStats software (http://www.empowerstats.com, X&Y solutions, Inc. Boston MA, USA) and R software (version 4.1.0, http://www.r-project.org) were used to analyze data and plot graphs. LASSO logistic regression analysis was conducted using the glmnet package in R. Nomogram plots were established by the root mean squares (RMS) package. The pROC and survival-ROC packages were applied to analyze ROC and time-dependent ROC (tROC) curves. Independent sample *t*-tests or Mann-Whitney *U*-tests were used to compare continuous variables, and chi-square tests were used to compare categorical variables. Statistical significance was defined as a *p*-value < 0.05.

## RESULTS

### Identification of the DEGs and the hub markers associated with prognosis in the training set

**Figure 2:**
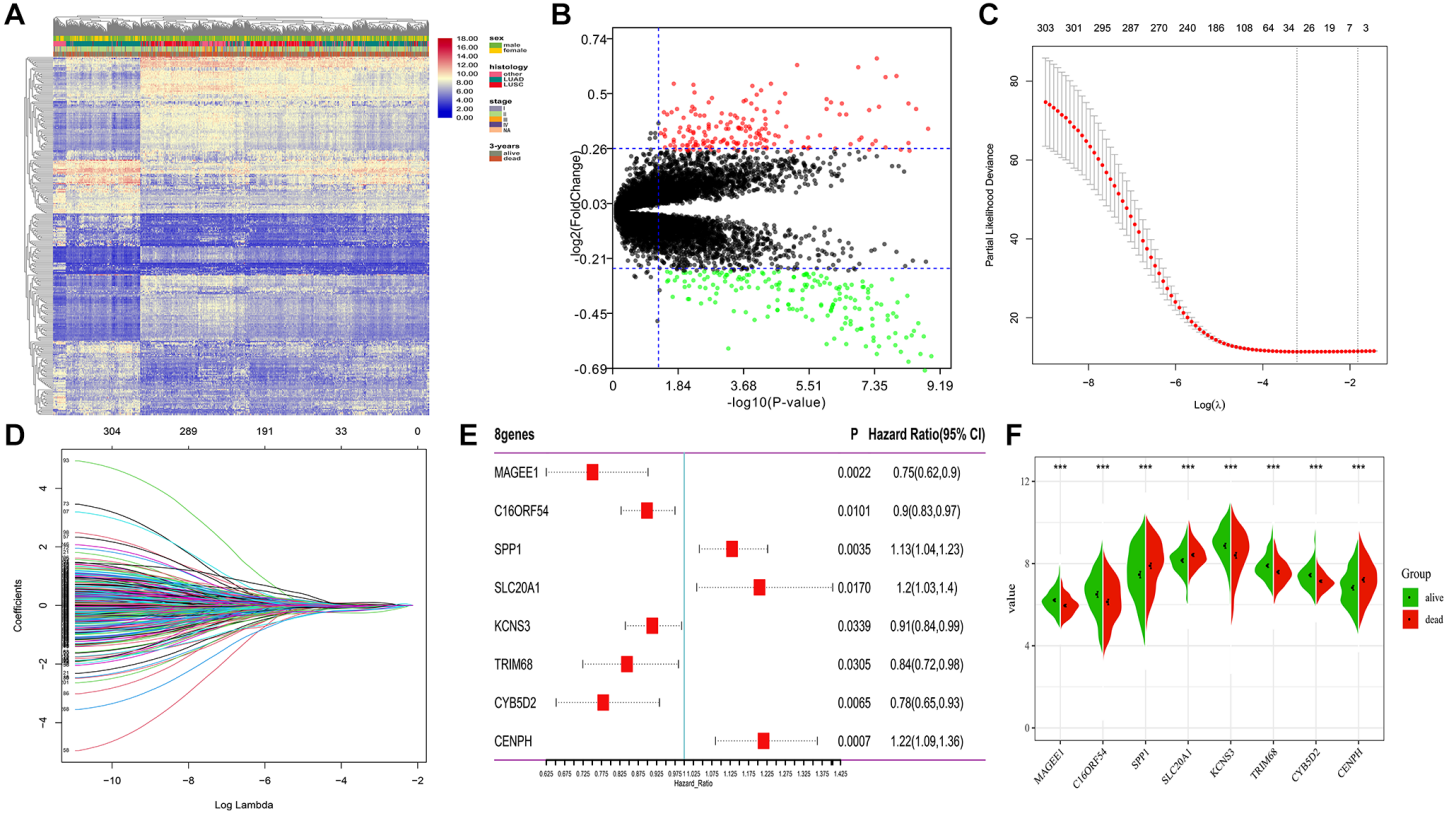
**Differential expression and LASSO-Cox regression results of DEGs.** (**A**, **B**) Heatmap plot and volcano plot represents the expression of 306 DEGs between samples from alive and deceased patients based on 3-year survival data, satisfying the criteria of adjusted *p*-value < 0.05, |log2FoldChange| >0.26. (**C**, **D**) 26 genes considered the more correlated with prognosis were identified by LASSO regression method. (**E**) Coefficients of eight genes estimated by multivariate Cox regression. (**F**) Expression profiles of eight hub genes between samples from alive and deceased patients with 3-year survival data.

A total of 651 lung cancer samples with the OS time over 30 days were analyzed. The LIMMA software was used to identify the DEGs between samples from alive or deceased patients with 3-year survival in lung cancer. Out of 14,052 genes, 306 met the threshold set (adjusted *p*-value < 0.05, |log2FC| >0.26), with 146 DEGs being down-expression and 160 being up-expression in the deceased group (Figure 2A, 2B).

To determine the most robust prognostic indicators, LASSO-Cox regression models were used. To overcome over-fitting, tenfold cross-validation was used on the 306 DEGs associated with 3-year survival. The more robust prognostic candidates were investigated using the LASSO regression method with an optimal value of 0.1618 (Figure 2C). The results showed that all 26 prognosis-related candidates had non-zero LASSO coefficients (Figure 2D). Subsequently, multiple stepwise Cox regression was used to determine the impact of the candidate genes, and eight hub markers were chosen to construct the risk model in lung cancer patients (Table 1). The expression profiles of these eight genes showed that elevated expression of secreted phosphoprotein 1 (SPP1), sodium-dependent phosphate transporter 1 (SLC20A1), and centromere protein H (CENPH) in lung cancer samples were risk factors for prognosis. In contrast, high-expression of MAGE family member E1 (MAGEE1), chromosome 16 open reading frame 54 (C16ORF54), potassium voltage-gated channel subfamily S member 3 (KCNS3), tripartite motif containing 68 (TRIM68) and cytochrome B5 domain containing 2 (CYB5D2) were protective factors for prognosis (Figure 2E). In addition, the expression of the eight genes was significantly different between samples from alive or deceased patients with 3-year survival in lung cancer (Figure 2F). The K-M survival curve analysis revealed that the expression of these eight genes is significantly associated with lung cancer prognosis (Supplementary Figure 1).

**Table 1:** The eight hub markers identified distinguish alive from deceased patients based on 3-year survival data.

**Gene symbol**	**Protein name (UniProt accession)**	**Log2FC (dead/alive)**	**Known functions**	**Relation to cancer**
SPP1	secreted phosphoprotein 1	0.43	Functions to control the survival, growth, differentiation and effector function of tissues and cells	Implicated in tumorigenesis in various cancer types [16, 17] Over expressed in lung neoplasms [18, 19]
SLC20A1	Sodium-dependent phosphate transporter 1	0.27	High-affinity inorganic phosphate:sodium symporter activity	High expression of SLC20A1 mRNA inhibits the progress of lung cancer [20]
KCNS3	Potassium voltage-gated channel subfamily S member 3	−0.47	Involved in energy metabolism	The expression is related to breast cancer and lung cancer, promoted metastasis [21, 22]
TRIM68	Tripartite motif containing 68	−0.31	Enhances the transcriptional activity of the AR [23]	Preferentially expressed in prostate cancer cells [23]
CYB5D2	Cytochrome b5 domain containing 2	−0.29	Interacting selectively and non-covalently with heme	Downregulation of CYB5D2 is associated with breast cancer progression [24]
CENPH	Centromere protein H	0.39	Negative regulation of cysteine-type endopeptidase activity	Higher expression levels of CENPH tended to have worse OS in lung cancer [25]
MAGEE1	MAGE family member E1	−0.26	Participating in specific biological processes	The expression of MAGEE1 is correlated with tumor-cell proliferation of NSCLC [26]
C16ORF54	Chromosome 16 open reading frame 54	−0.37	Protein amino acid binding and integral component of membrane	Tobacco Smoke Pollution results in decreased expression of C16ORF54 mRNA in lung cancer [27]

### Construction and validation of the PRS in lung cancer

The predictive model was constructed using the eight hub markers identified using the multiple Cox regression method. The risk score of each patient was calculated based on the cox coefficients: PRS = 0.1223 × expression level of SPP1 + 0.1862 × expression level of SLC20A1 – 0.0909 × expression level of KCNS3 –0.1693 × expression level of TRIM68 – 0.2490 × expression level of CYB5D2 + 0.1954 × expression level of CENPH – 0.2869 × expression level of MAGEE1 – 0.1069 × expression level of C16ORF54.

**Figure 3:**
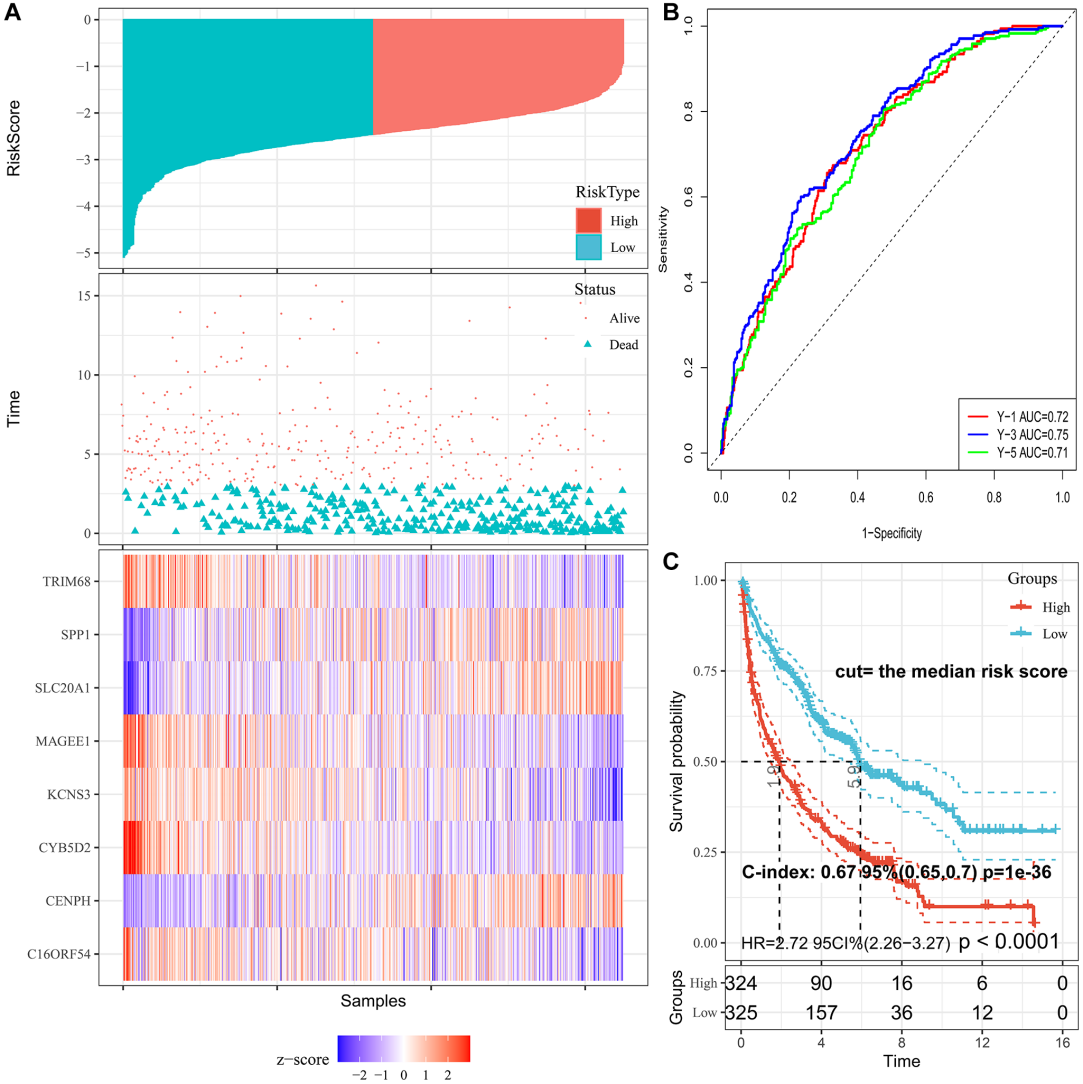
**Characteristics of PRS signature in the training cohort.** (**A**) Risk scores distribution, survival status, and gene expression patterns of patients in high- and low-risk groups in the training cohort. (**B**) Time-dependent ROC analysis for predicting OS. (**C**) Survival curves and C-index for high- and low-risk groups.

The cut-off value was determined automatically based on the median risk score, and lung cancer patients were divided into the low- (*n* = 390) and high-risk (*n* = 259) groups using the cut-off value of −2.39. As illustrated in Figure 3A, the distribution of the PRS, OS time, and heatmap for the eight-gene signature in the training set is shown from top to bottom. Furthermore, the tROC analysis revealed that PRS was the most accurate predictor of OS (Supplementary Figure 2). The AUC of this PRS model in 1-year, 3-year, 5-year was 0.72, 0.75, and 0.71, respectively (Figure 3B). Moreover, the K-M survival analysis revealed that the patients had worse OS in the high-risk group than in the low-risk group (HR = 2.72; 95% confidence interval (CI), 2.26 to 3.27, *p* < 0.0001), and the C-index of the PRS for predicting survival was 0.67; 95% CI, 0.65 to 0.70 (Figure 3C).

### Validation of PRS signature in the subgroup and independent lung cancer validation sets

**Figure 4:**
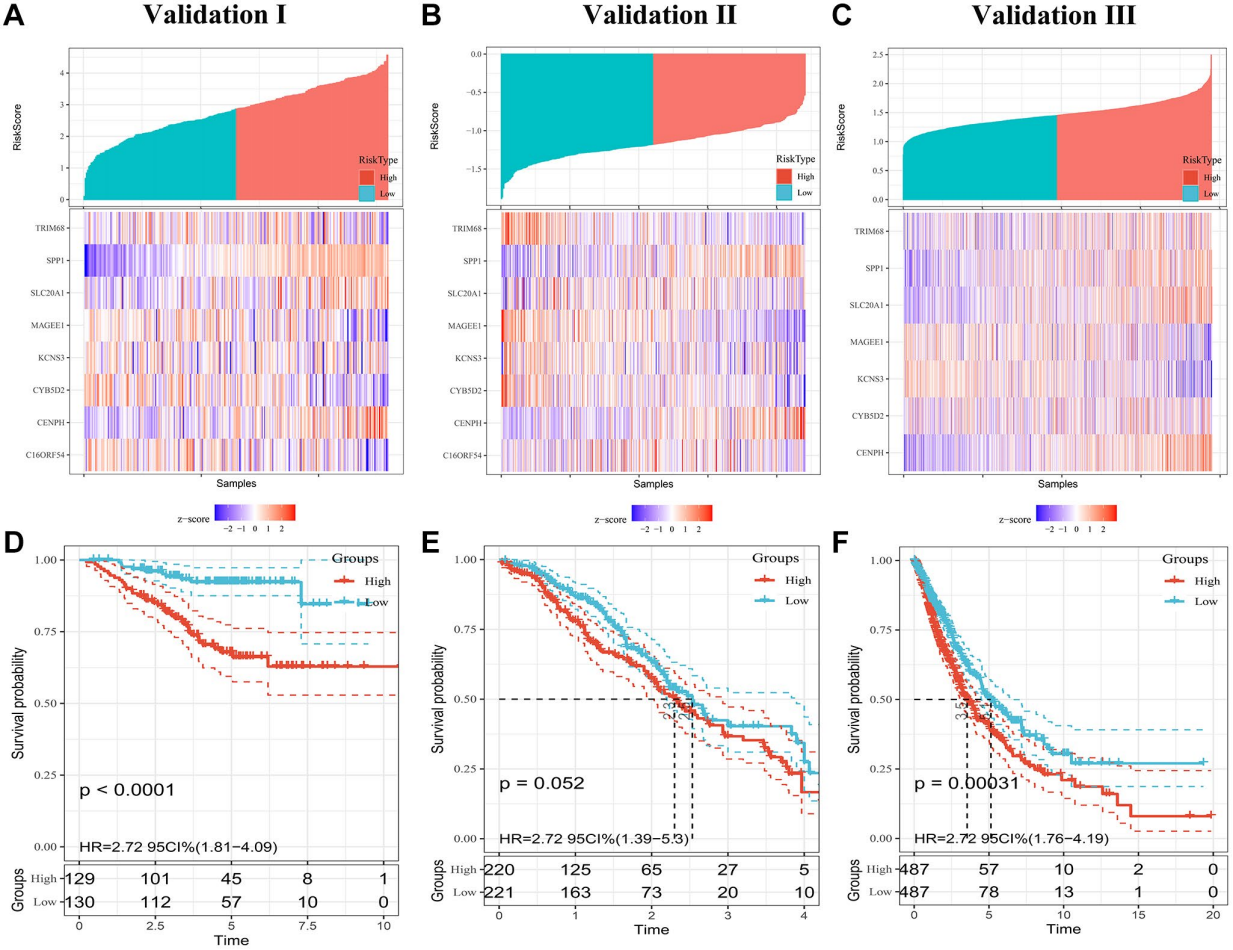
**Evaluating PRS signatures in validation sets.** (**A**–**C**) Risk score distribution and survival status of patients in high- and low-risk groups in validation sets. (**D**–**F**) Survival curves in validation sets.

To confirm the prognostic robustness of PRS features and complex models across cohorts, we further validated it in the three independent external cohorts described earlier. Similarly, in each of the three validation sets, patients in the high-risk group had poorer outcomes, while those in the low-risk group had a higher survival rate (Figure 4A–4C). K-M analysis confirmed that the predicted high-risk group had a significantly shorter time to death (Figure 4D–4F), indicating good predictive performance of the PRS, with a HR of 2.38; 95% CI, 1.61 to 3.53 in validation set I, 1.35; 95% CI, 1.06 to 1.71 in validation set II, and 2.71; 95% CI, 1.77 to 4.18 in validation set III.

**Figure 5:**
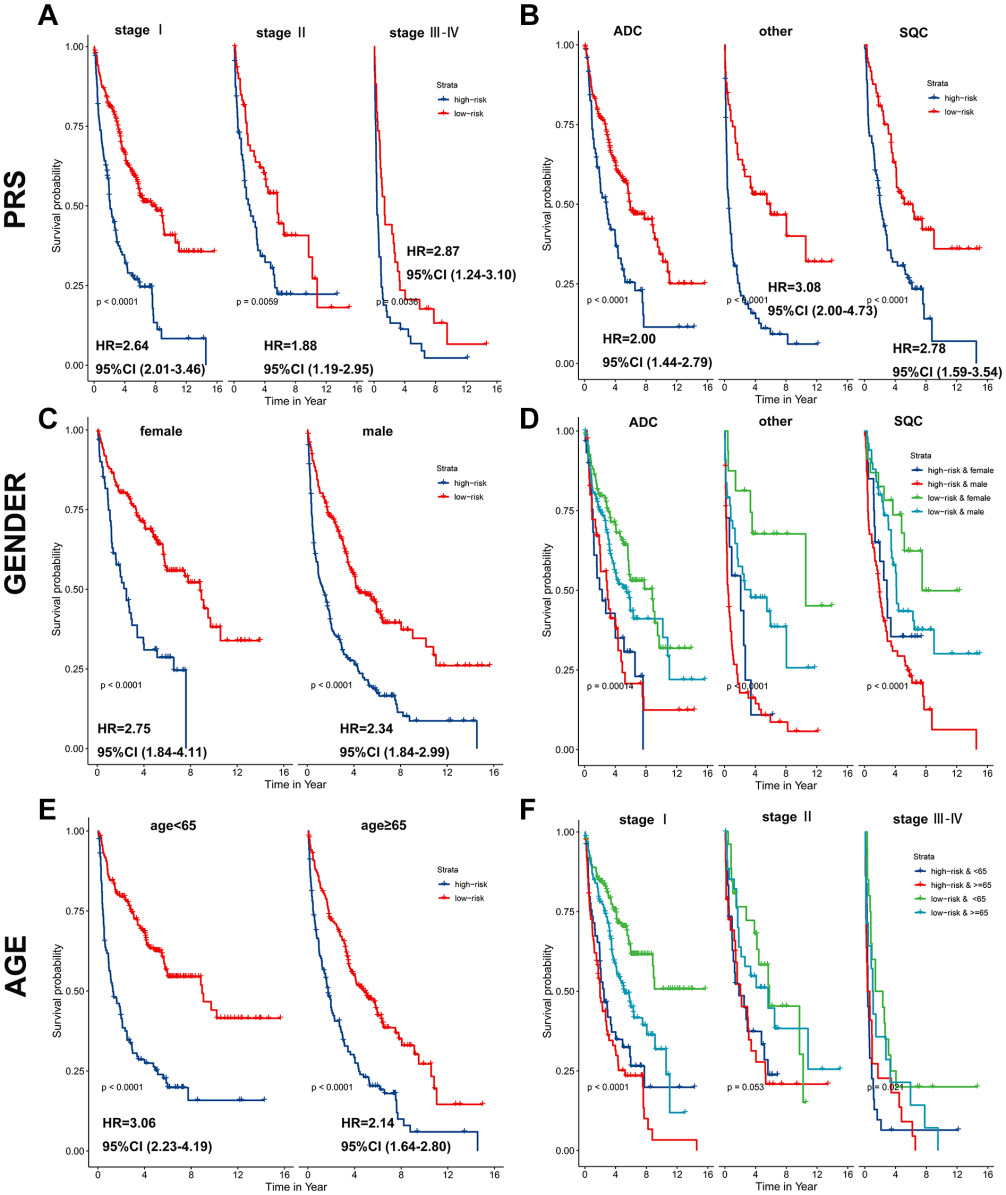
**PRS as a valuable predictor for OS in subgroups.** PRS discriminated high-risk patients with different clinicopathological characteristics, including (**A**) p-stage, (**B**) histological type, (**C**, **D**) gender, and (**E**, **F**) age.

Subsequently, a stratified analysis was performed to assess whether PRS characteristics could predict the probability of patient survival in the same subgroup of clinical factors. Patients in the training cohort were classified clinically by p-stage (I/II/III-IV) (Figure 5A), histological type (glandular/squamous) (Figure 5B), gender (female/male) (Figure 5C, 5D) and age (<65/≥65) (Figure 5E, 5F). The results showed that PRS characteristics could divide patients with the same age, sex, p-stage, and histological type into high-risk and low-risk groups. In each tier, OS was shorter in patients with high-risk scores than in patients with low-risk scores (*p* < 0.001) (Figure 5). As a result of the above analysis, the HR does not change significantly across subgroups, and the PRS is an independent risk factor of lung cancer prognosis. It has predictive value in different people. In addition, to further prove the specificity of PRS as a prognostic factor of lung cancer in the clinic, we tested it in four other primary global cancers. The results showed that PRS signature was not associated with the prognosis of liver cancer, bowel cancer, gastric cancer, or breast cancer (Supplementary Figure 3), indicating that the PRS signature is only related to the prognosis of lung cancer.

### Prognostic nomogram for OS

**Figure 6:**
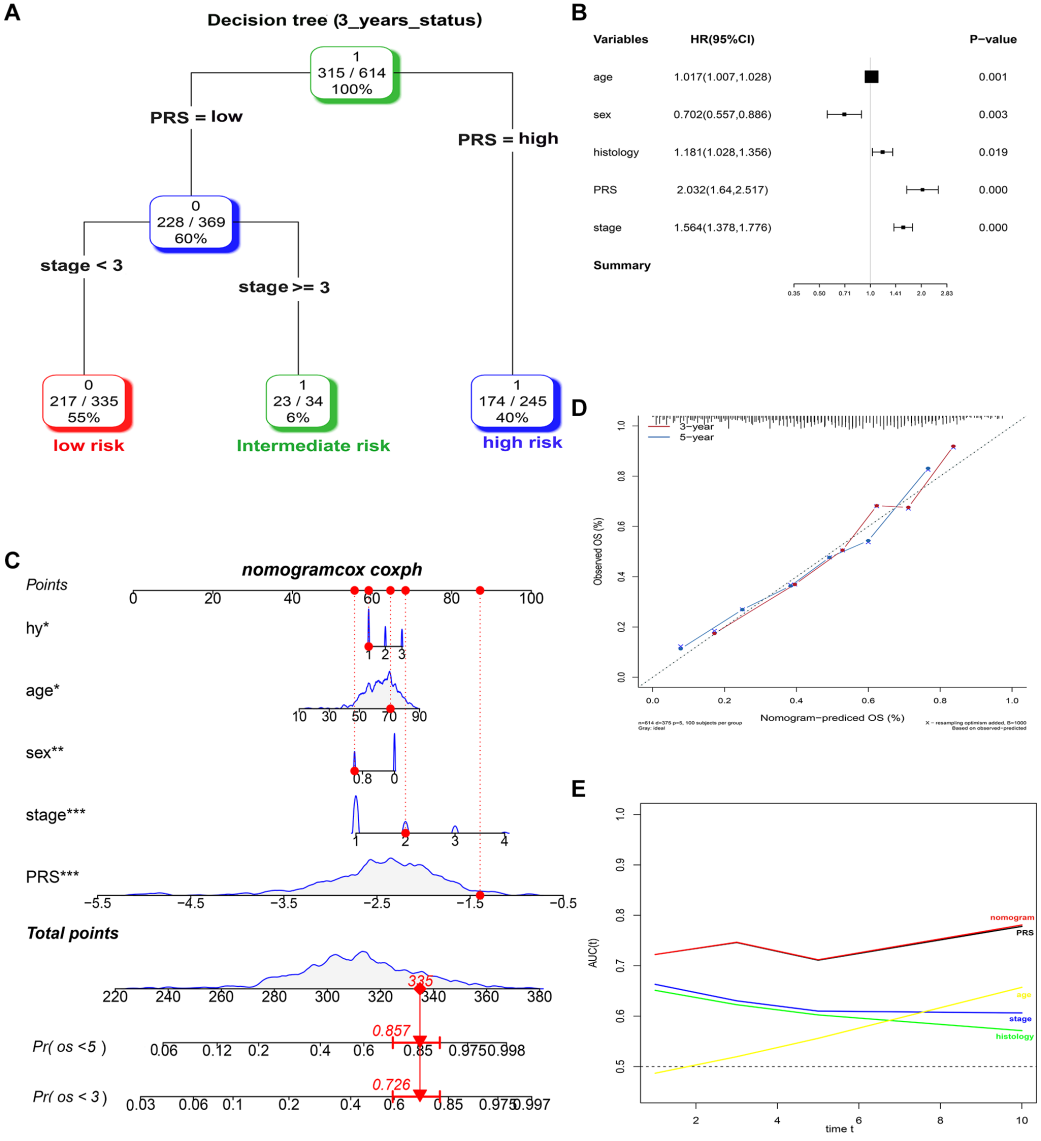
**Combination of the PRS signature and clinical features improves survival prediction in training sets.** (**A**) A decision tree was constructed to improve risk stratification. (**B**) Multivariate Cox regression model (complex model). (**C**) Survival nomogram for quantifying risk assessment for individual patients. (**D**) Calibration analysis revealed a high degree of accuracy in predicting survival at 3 or 5 years. (**E**) Among all clinical variables, tROC analysis demonstrated that the nomogram was the most stable and powerful predictor of OS.

A total of 614, 259, 438, and 740 patients with full-scale five clinical annotations including age, sex (male or female), histology (LUAD, LUSC or other), p-stage (I, II, III or IV) and PRS (low or high) were extracted from the training, validation I, II, and III sets, respectively (Table 2). In the training set, multivariate Cox regression analysis revealed that these five variables were correlated with the prognosis of lung cancer, with PRS being the most significant predictor of overall survival in the Cox model (complex model) (Figure 6A). These variables were used to construct a decision tree to improve risk stratification for overall survival. As shown in Figure 6B, only p-stage and PRS remained in the decision tree, with three different risk subgroups identified. To quantify the risk assessment and survival probability for individual patients, a nomogram incorporating PRS and other clinicopathological features was constructed (Figure 6C). Furthermore, we calculated the value of each covariate of patient No. 350 (GSM1213824) and mapped it to the corresponding score, calculated the total score, and its probability at 3-year and 5-year survival. The calculated values were 0.757 and 0.881. For 3- or 5-year survival, the probability calibration plot revealed the best agreement between nomogram prediction and actual observation (45-degree dotted line) (Figure 6D), indicating that the nomogram is highly accurate. When compared to other features, the nomogram exhibited the most powerful and stable ability for survival prediction, with an average AUC greater than 0.7, significantly better than the pathological p-stage (Figure 6E).

**Table 2:** Clinical characteristics and PRS model of lung cancer patients in the training and validation sets.

**Exposure**	**Training set**	**Validation set I**	**Validation set II**	**Validation set III**
	***N* = 614**	***N* = 259**	***N* = 438**	***N* = 740**
Age	64.29 ± 10.55	60.4 ± 7.9	65.0 ± 9.9	66.1 ± 9.5
Gender	–	–	–	–
Male	404 (65.8%)	133 (51.4%)	230 (52.5%)	453 (61.2%)
Female	210 (34.2%)	126 (48.6%)	208 (47.5%)	287 (38.8%)
Histology	–	–	–	–
LUAD	308 (50.2%)	234 (90.3%)	309 (70.5%)	476 (64.3%)
LUSC	164 (26.7%)	25 (9.7%)	120 (27.4%)	264 (35.7%)
Other	142 (23.1%)	0	9 (2.1%)	0
P-stage	–	–	–	–
I	402 (65.5%)	186 (71.8%)	237 (54.1%)	383 (51.8%)
II	125 (20.4%)	56 (21.6%)	81 (18.5%)	192 (26.0%)
III	77 (12.5%)	17 (6.6%)	114 (26.0%)	135 (18.2%)
IV	10 (1.6%)	0	6 (1.4%)	30 (4.0%)
PRS	–	–	–	–
Low-risk	369 (60.0%)	129 (49.8%)	220 (49.8%)	487 (50.0%)
High-risk	245 (40.0%)	130 (50.2%)	221 (50.2%)	487 (50.0%)

At the same time, the complex model combining PRS, and clinical risk factors also had a good predictive performance of 3-year survival, namely 0.788, 0.709 and 0.614, respectively in the three validation sets (Supplementary Figure 4).

### Functional analysis of the survival-related DEGs

**Figure 7:**
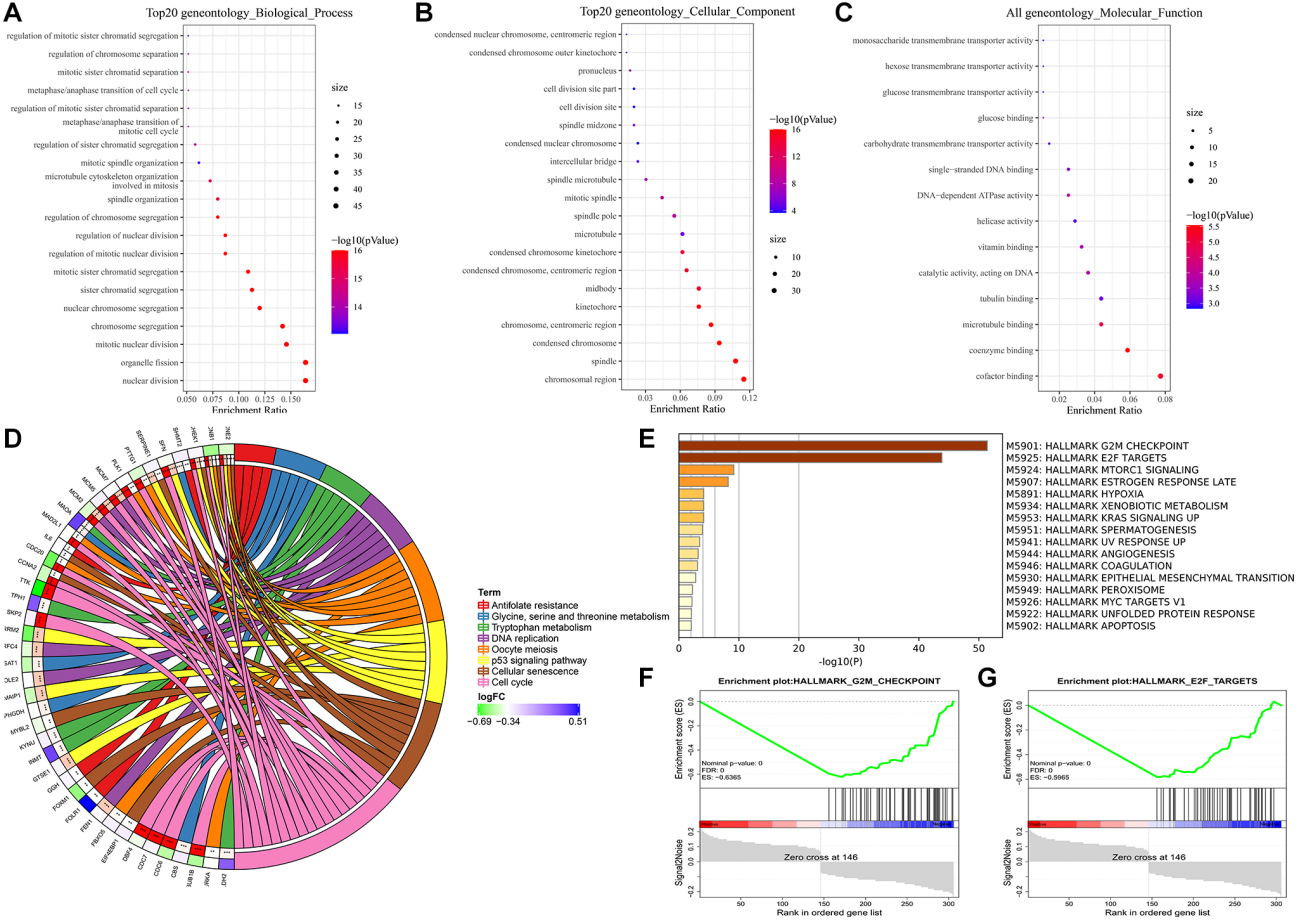
**Enrichment analyses of DEGs.** (**A**) Biological process. (**B**) Cellular component. (**C**) Molecular function. (**D**) KEGG pathway analysis. (**E**–**G**) GSEA analysis using hallmark gene sets from MSigDB.

**Figure 8:**
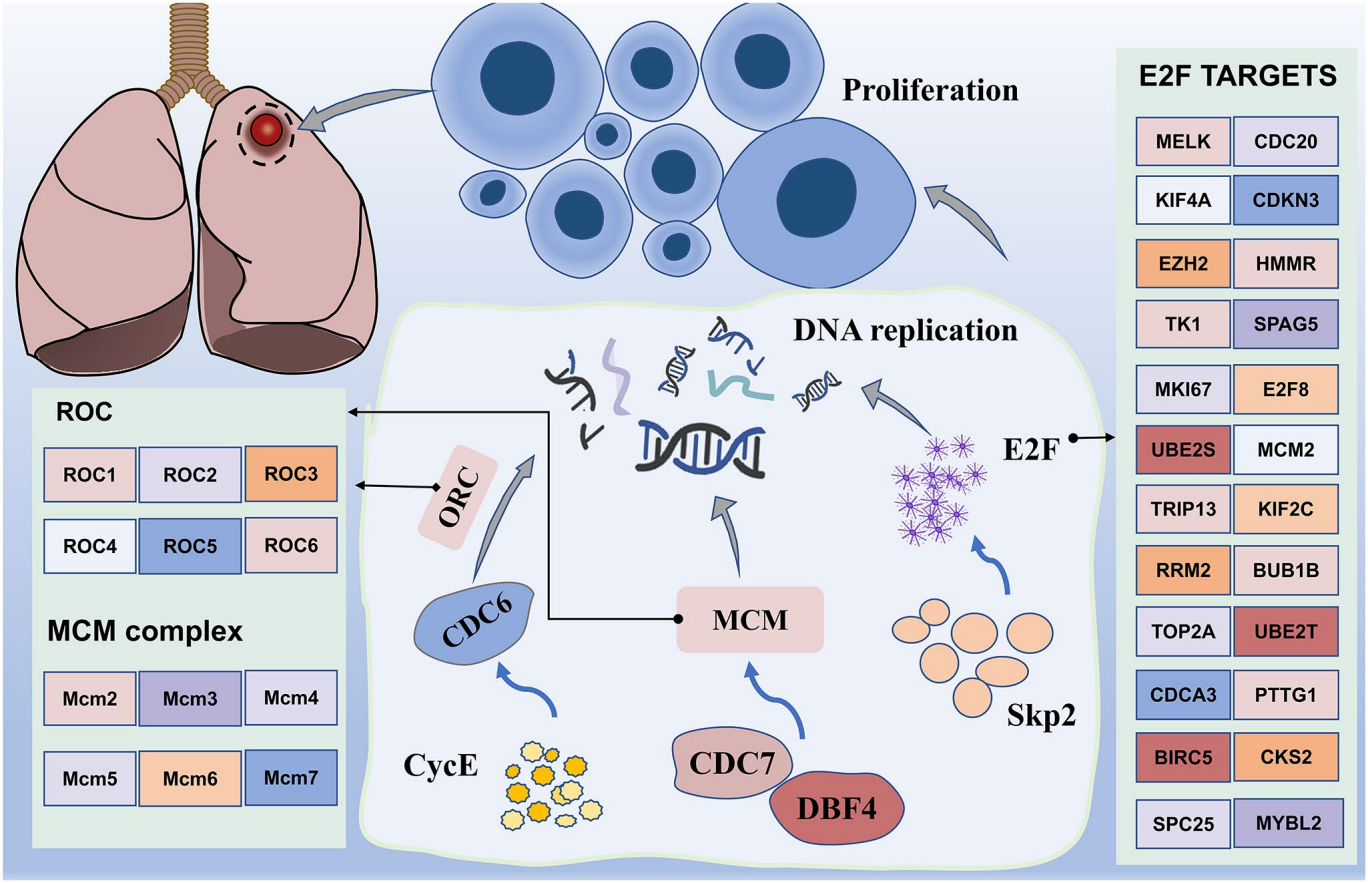
**Working model of major enrichment pathways in lung cancer.** Proteins activated by CDC7, DBF4, CYCE, MCM, and CDC6 promote DNA replication, in addition to promoting cell amplification, particularly transcription factor E2F, which is regulated by numerous genes. In addition, upregulation of the transcription activity of E2F promotes Skp2 regulation of the PI3K/AKT pathway, thereby potentially promoting the occurrence of lung cancer.

To further understand the underlying mechanism of the survival-related DEGs, we analyzed 306 DEGs between samples from alive or deceased patients with 3-year survival in the training set. Enrichment analyses involved the KEGG, GO functional enrichment and GSEA of hallmark in MSigDB. The genes were divided into 3 categories: biological process, cellular component, and molecular function according to the GO terms (Figure 7A–7C). The most abundant groups were nuclear division, chromosomal region, and cofactor binding, respectively, in the three categories. We discovered that pathways involving the cell cycle, cellular senescence, oocyte meiosis, and the p53 signaling pathway were enriched in KEGG (Figure 7D). Additionally, the GSEA results based on the hallmark gene sets in MSigDB indicated that these DEGs were primarily associated with not only HALLMARK G2M CHECKPOINT (normalized enrichment score (NES) = −1.30, *p* < 0.001), but also HALLMARK E2F TARGETS (NES = 2.024, *p* < 0.001) (Figure 7E–7G). The top pathway and hallmark:cell cycle and E2F TARGETS clarified the division of activity in lung cancer cells. We summarized a working model of the activated pathways in Figure 8.

## DISCUSSION

Lung cancer, one of the most common malignant tumors worldwide, claims over one million deaths each year and has a dismal 5-year survival rate [28]. Thus, changes in the prognosis of lung cancer patients may occur long before detectable clinicopathological abnormalities, highlighting the correlation between biomarkers such as the expression of specific genes (i.e., hub genes) and lung cancer prognosis [29, 30]. Many studies at the biological and clinical levels have suggested the link between gene mutation sites and disease progression, and the use of high-throughput sequencing data based on omics to make more accurate diagnosis and prognosis predictions for lung cancer patients, so as to formulate individualized treatment plans on this basis to bring greater benefits to the prognosis of patients [31].

The current cancer progression prediction is mainly based on disease manifestations and the Tumor-Node-Metastasis staging system of American Joint Commission on Cancer (AJCC). However, both methods' static representations of clinicopathologic factors fail to account for the genetic heterogeneity of cancer, limiting their predictive value [11]. Recent studies have shown that gene mutations and expression disorders are associated with disease progression and therapeutic response in lung cancer [32–34]. However, these current biomarkers, such as epidermal growth factor receptor (EGFR) and Kirsten rat sarcoma virus (KRAS) oncogene homologs, do not fully represent the complex mechanisms of lung cancer progression [35, 36]. Larsen et al. developed a 54-gene signature in LUAD [10], but the combined accuracy in predicting recurrence is only 69% (79% sensitivity, 59% specificity). Sheng et al. reported a new biological marker discovery pathway, which integrates RNA sequencing (RNA-seq) and clinical data to identify progression gene signatures (PGSs) based on survival genes, and discovered 22 LUAD-PGS genes and 23 LUSC-PGS genes that have a high predictive value (area under the curve (AUC) = 0.85, 0.92, respectively) [11]. This model needs to be further optimized to facilitate clinical implementation. Recently, Xie et al. developed a prognostic model for death due to extensive-stage squamous cell lung cancer (SCLC), with an unadjusted concordance (C)-index of 0.714 [12].

In this study, through more rigorous identification, identified eight independent prognostic genes to establish a risk score for assessing the survival probability in the training set. As shown in Table 1, several of the gene signatures we identified have been investigated in various types of tumors. For example, MAGEE1 was associated with important clinical and molecular features in glioma [37, 38], and can be considered an important marker in determining the prognosis of glioblastoma [39]. More importantly, the expression of MAGEE1 is correlated with tumor-cell proliferation of NSCLC [26]. As a gene that is overexpressed in breast, bladder, colorectal, head and neck, liver, lung, and esophageal cancers [40], SPP1 has the potential to influence not only the occurrence and progression of LUAD, but also to serve as an independent prognostic marker and a novel therapeutic target [41–43]. High SLC20A1 expression is associated with poor prognoses in basal-like breast cancers, longue cancer and esophageal adenocarcinoma [44–46]. CENPH was found to drive the molecular changes during the pathologic stages of LUAD [47], and patients with a higher expression level of CENPH tended to have a poorer OS [25]. We constructed PRS signatures containing these eight genes under a novel pipeline to support prognosis and OS prediction of lung cancer. The 8-gene PRS and the complex model both had predictive effects in three large cohorts, with AUCs exceeding 70% or even 80%. Taken together, these results suggest that the variable expression of 8-gene model is associated with different prognosis in lung cancer, and may serve as a prognostic biomarker as well as a treatment target for lung cancer patients.

The prognostic value of PRS features was further validated in another two independent sets. PRS was able to identify high-risk patients in both validation groups, implying that it can be used as a reliable risk factor for the overall population. Patients with higher PRS had poorer survival compared to those with lower PRS. In addition, when combined with clinical risk factors, a column line plot was built for the risk quantification in individual patients. ROC analysis showed that PRS had considerable risk predictive power for OS, and calibration analysis showed that nomogram survival prediction results were extremely close to actual survival.

In addition, we note the enrichment results, which mainly include the cell cycle. Interestingly, the GSEA results from the hallmark gene sets were also enriched for genes encoding cell cycle-related targets of the E2F transcription factor. Our data uncovered differential expression of multiple genes, including CDC6, CDC7, DBF4, MCM and CycE (Figure 8). Most of them may induce SQLC through DNA replication and cell cycle pathway [48]. Minichromosome maintenance complex component 4 (MCM4), a highly expressed gene in NSCLC, is required for the proliferation of NSCLC cells [49]. MCM proteins, including MCM2-7, are also required for replication initiation and elongation [50]. In addition, Skp2 activated PI3K/AKT pathway activities by upregulating the transcription activity of E2F, thereby potentially promoting the occurrence of lung cancer [51]. These results suggest that the transcriptional regulation through E2F may be a novel therapeutic target in lung cancer.

Although our study reveals the feasibility of a new approach to biomarker discovery that integrates cancer survival and overall genetic profiling data, important questions remain to be addressed in order to facilitate the clinical implementation of PRS in clinical prognostic testing. Our data demonstrate the feasibility of using PRS as a clinical test; however, large-scale clinical studies are needed to statistically validate the ability of PRS to define patients at high risk for poor prognosis. Future studies will also aim to develop new companion therapies for PRS and other biomarker discovery pipelines.

## CONCLUSIONS

To predict OS in patients with lung cancer, we constructed an eight-gene based PRS that was further validated in another three validation sets as well as other cancer sets. Because PRS was found to be associated with independent and specific risk factor for lung cancer, patients with higher PRS had poorer survival outcome. By combining genes signature with clinical features, we developed a nomogram model to quantify the risk for individual patients. This model can also be used to identify patients who may benefit from adjuvant therapy, allowing for more personalized treatment in lung cancer. In addition, enrichment analysis revealed that the key genes were associated with the cell cycle and E2F targets.

## Supplementary Materials

Supplementary Figures

Supplementary Table 1
