# Supplementary material for: Construction and evaluation of a nomogram for predicting survival in patients with lung cancer
Source: Aging (Albany NY). 2022 Mar 23;14(6):2775–92. doi: 10.18632/aging.203974 (PMC9004553; doi:10.18632/aging.203974)
Supplement: Supplementary Figures [file aging-14-203974-s001.pdf]

SUPPLEMENTARY FIGURES

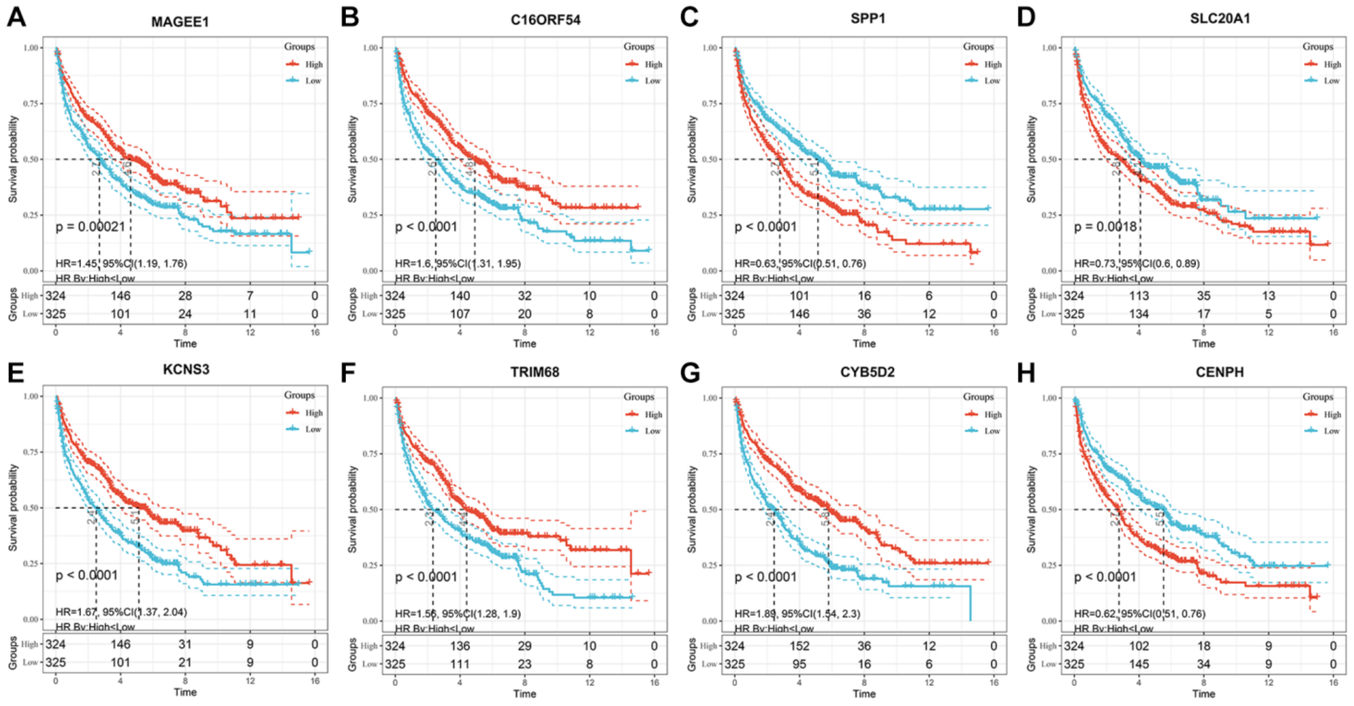

Supplementary Figure 1. The K-M survival curve analyses of 8-genes. (**A**) MAGEE1; (**B**) C16ORF54; (**C**) SPP1; (**D**) SLC20A1; (**E**) KCNS3; (**F**) TRIM68; (**G**) CYB5D2; (**H**) CENPH.

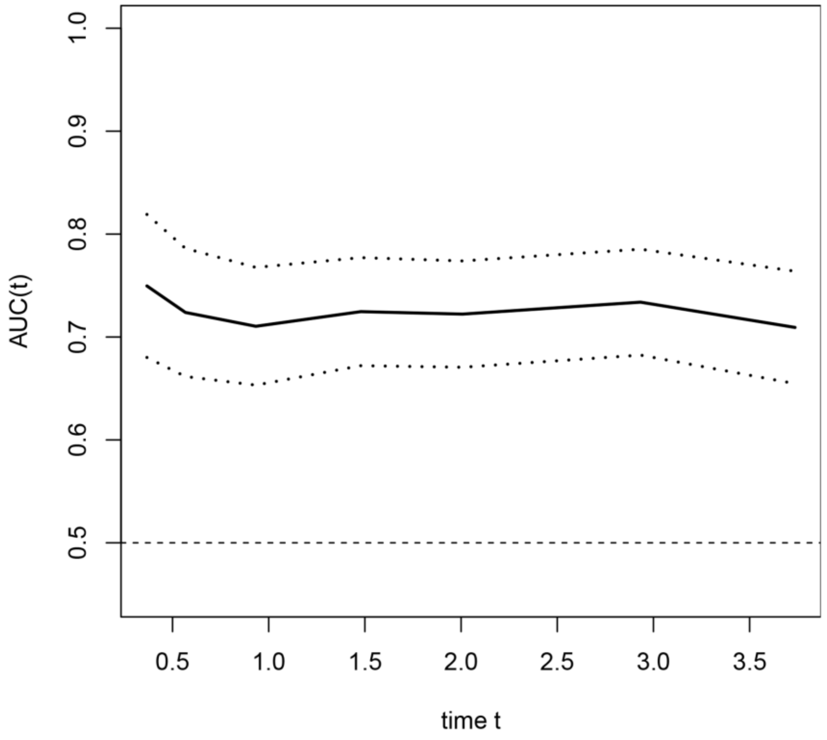

Supplementary Figure 2. The tROC analysis of the PRS.

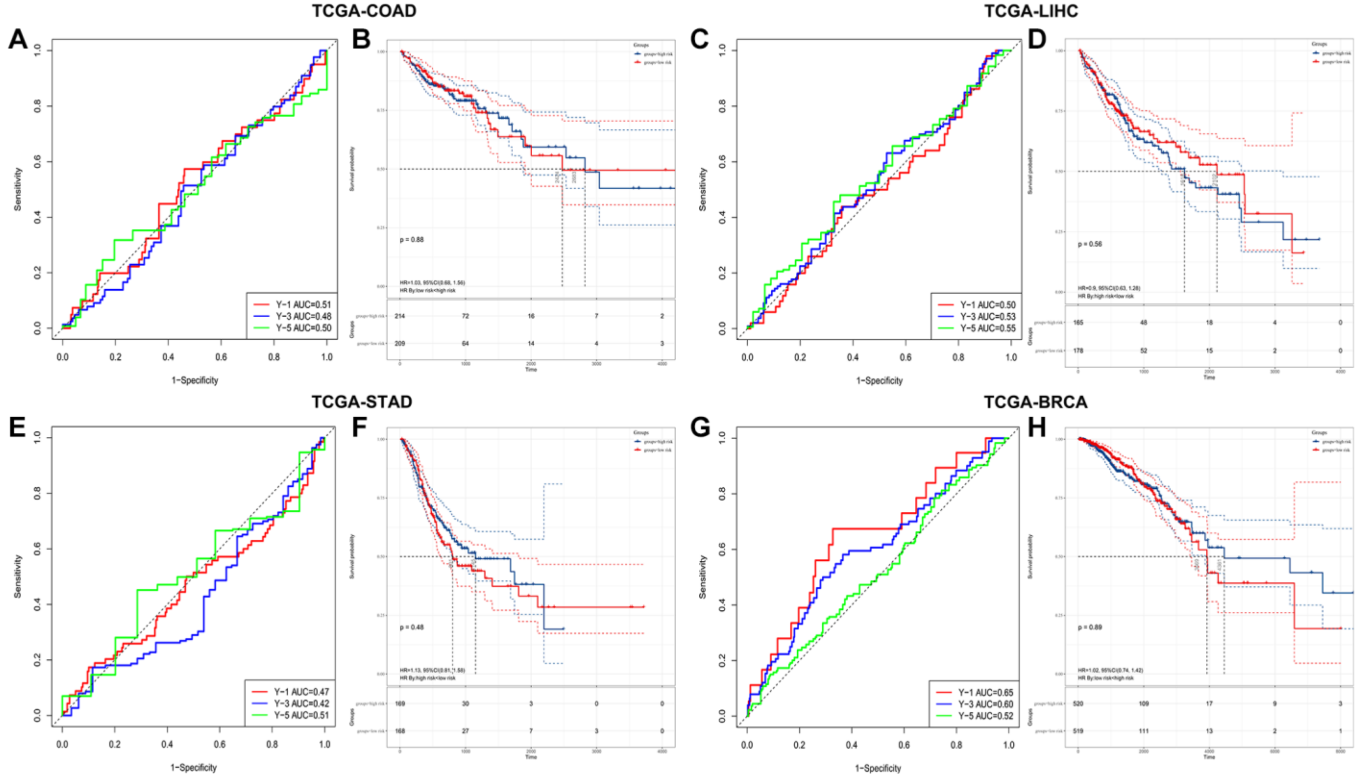

Supplementary Figure 3. The PRS as a valuable predictor for OS in other cancers. (**A**, **B**) TCGA-COAD; (**C**, **D**) TCGA-LIHC; (**E**, **F**) TCGA-STAD; (**G**, **H**) TCGA-BRCA.

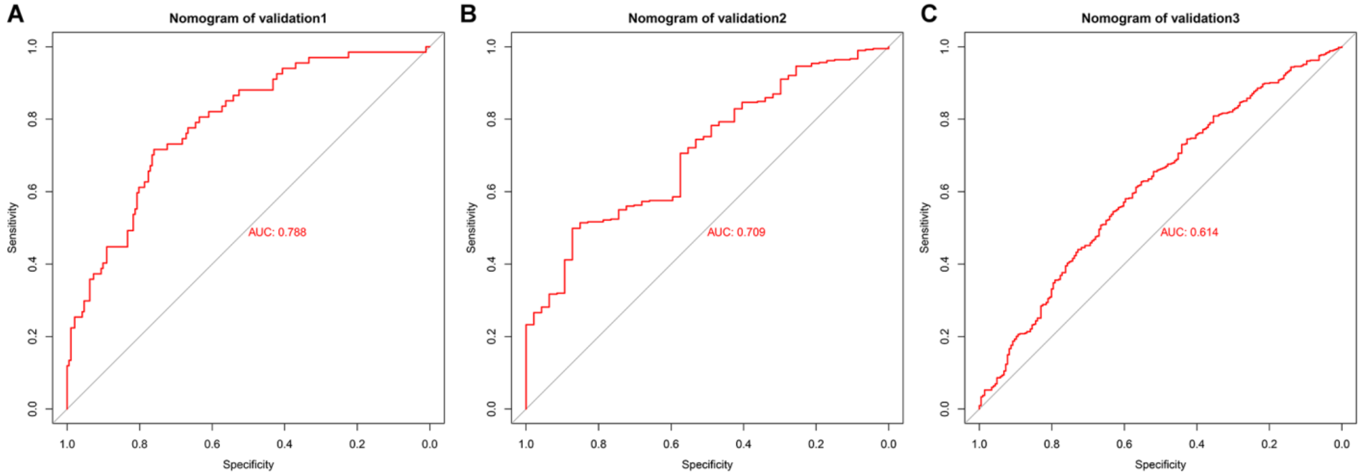

Supplementary Figure 4. ROC analysis of the complex model in validation sets. (**A**) the validation set I; (**B**) the validation set II; (**C**) the validation set III.
