# Supplementary material for: Construction and evaluation of a nomogram for predicting survival in patients with lung cancer
Source: Aging (Albany NY). 2022 Mar 23;14(6):2775–92. doi: 10.18632/aging.203974 (PMC9004553; doi:10.18632/aging.203974)
Supplement: Supplementary Table 1 [file aging-14-203974-s002.pdf]

SUPPLEMENTARY TABLE

Supplementary Table 1. Information on the datasets in the training and validation sets.

Cohorts	Datasets	Year	Country	Sample	*N*
Training set	GSE30219	2011	France	lung cancer	274
	GSE37745	2012	Sweden	NSCLC	196
	GSE50081	2013	Canada	NSCLC	181
Validation set I	GSE29013	2011	USA	NSCLC	55
	GSE31210	2011	Japan	LUAD	204
Validation set II	GSE41271	2012	USA	lung cancer	268
	GSE42127	2012	USA	NSCLC	173
Validation set III	TCGA-LUAD	2014	USA	LUAD	494
	TCGA-LUSC	2014	USA	LUSC	480
Specificity validation set	TCGA-LIHC	2015	USA	LIHC	343
	TCGA-COAD	2015	USA	COAD	423
	TCGA-STAD	2015	USA	STAD	337
	TCGA-BRCA	2015	USA	BRCA	1039
